# Analysis of Galectin-3 in Differentiating Non-malignant and Malignant Nodular Thyroid Lesions

**DOI:** 10.7759/cureus.102189

**Published:** 2026-01-24

**Authors:** Suganya J, Keerthikasri ES, Sowmya Srinivasan

**Affiliations:** 1 Pathology, Mahatma Gandhi Medical College and Research Institute, Sri Balaji Vidyapeeth (SBV) University, Puducherry, IND

**Keywords:** biomarker, diagnosis, galectin-3, immunohistochemistry, immunosurveillance, targeted therapy, thyroid cancer

## Abstract

Introduction

Accurate differentiation of benign and malignant thyroid nodules is critical for appropriate management. Fine-needle aspiration cytology (FNAC), although valuable, has limitations, especially in follicular-patterned neoplasms. Galectin-3, a beta-galactoside-binding lectin involved in cell proliferation and apoptosis, has been proposed as a diagnostic immunohistochemical marker. This study evaluates the utility of galectin-3 in distinguishing malignant from non-malignant nodular thyroid lesions.

Methods

A prospective observational study was conducted on 56 female patients undergoing thyroidectomy. Histopathological evaluation categorized cases into the benign (n=25), borderline (n=6), and malignant (n=25) groups. Immunohistochemical staining for galectin-3 was performed on paraffin-embedded tissue sections. Positivity was defined by cytoplasmic and/or nuclear staining.

Results

Galectin-3 expression was observed in 66.7% (14/21) of malignant lesions, predominantly in classic papillary thyroid carcinoma. None of the benign or borderline cases exhibited galectin-3 positivity. Notably, other papillary carcinoma variants were also negative. Statistical analysis showed a significant correlation between galectin-3 expression and malignancy (p < 0.001).

Conclusion

Galectin-3 is a highly specific immunohistochemical marker for malignant thyroid lesions, particularly classic papillary thyroid carcinoma. Its absence in benign and borderline lesions highlights its diagnostic value. Incorporating galectin-3 into routine histopathological evaluation can improve diagnostic accuracy, especially in indeterminate or follicular-patterned thyroid neoplasms.

## Introduction

The thyroid gland, located in the anterior neck, plays a crucial endocrine role by producing the hormones T3 and T4. These hormones regulate metabolism, influencing energy expenditure and heat production [1].

Thyroid nodules are common, with palpation detecting them in 4-7% of adults, while ultrasound reveals a prevalence of up to 67%. Although over 90% are benign, 4-15% may be malignant. Accurate evaluation is essential for proper management [2,3]. Histopathology is the gold standard for diagnosing thyroid neoplasms but poses challenges. Follicular-patterned lesions often show overlapping features, making benign-malignant differentiation difficult [4]. Limited biopsy samples may not reveal crucial capsular or vascular invasion. Inconsistent tissue representation can hinder accurate diagnosis. Additionally, subjective diagnostic criteria may lead to variability among pathologists [5].

Biomarkers play a crucial role in risk stratification and personalized management of thyroid lesions. Elevated salivary FT3, thyroglobulin (Tg), and calcitonin levels are associated with increased risk and aggressiveness of thyroid cancers such as papillary thyroid carcinoma (PTC) and medullary carcinoma [6]. These biomarkers aid in diagnosis, guide surgical decisions, and help monitor treatment response and recurrence. Establishing cut-off values enhances clinical decision-making, while predictive models integrating multiple biomarkers allow for individualized treatment planning and follow-up [7].

Galectin-3, a β-galactoside-binding protein implicated in diverse cellular functions, has garnered significant attention in thyroid pathology. It serves as a valuable adjunct to conventional diagnostic modalities [8]. Elevated galectin-3 expression within thyroid tissue is frequently observed in malignant neoplasms, particularly papillary thyroid carcinoma, compared to benign lesions [9]. Moreover, galectin-3 expression correlates with tumor aggressiveness, providing crucial prognostic information and guiding optimised treatment strategies [10].

Galectin-3 regulates tissue organization, wound healing, and immune responses through cell-cell and cell-matrix interactions. It promotes cell proliferation by activating pathways involved in cell division and cycle progression. While vital for normal tissue repair, this activity can also drive uncontrolled growth in cancers [11,12].

Galectin-3 demonstrates a complex role in apoptosis. While it can exhibit anti-apoptotic effects by interacting with survival proteins and inhibiting apoptotic signalling, it can also contribute to cell death under certain conditions. This multifaceted influence on cell fate underscores the significance of galectin-3 as a key regulator of cellular homeostasis and a potential target for therapeutic interventions [13,14].

Further research is needed to fully understand the role of galectin-3 and optimize its clinical application for consistent and reliable use. Hence, this study aims to investigate the diagnostic utility of galectin-3 in distinguishing between malignant and non-malignant thyroid nodules.

## Materials and methods

A total of 56 thyroid specimens of female patients with inclusion criteria of histopathologically diagnosed non-malignant and malignant nodular thyroid lesions were collected between January 2023 and April 2025 after obtaining Institutional Ethical Committee clearance from Mahatma Gandhi Medical College and Research Institute.

The retrospective samples fulfilling the inclusion criteria were taken from stored paraffin blocks from which sections were taken. For the prospective samples, paraffin blocks after histopathological diagnosis that fulfilled the inclusion criteria were obtained, and sections were taken.

These tissue sections were subjected to H&E stain to analyse the morphology, and IHC marker galectin-3. The clinical and demographic details, pre-operative diagnosis, and gross examination findings of all cases were retrieved from the hospital information system. All techniques and staining were done as per the standard operative procedures of our laboratory. Based on histopathological evaluation, the lesions were categorized into malignant and non-malignant groups. Galectin-3 expression was assessed through immunohistochemistry and correlated with the morphological classification.

Criteria for positivity

Tumor immunoreactivity was evaluated based on the percentage of positive tumor cells, staining intensity, and staining pattern. Cases were considered positive when more than 10% of tumor cells demonstrated cytoplasmic staining, while 1-10% positivity was reported as focal or weak staining, and less than 1% or complete absence of staining was regarded as negative. Staining intensity was graded semiquantitatively as 0 (none), 1+ (weak), 2+ (moderate), or 3+ (strong). Cytoplasmic staining in tumor cells was assessed, and moderate to strong intensity (2+-3+) was generally required to classify a case as positive. Weak (1+) or equivocal staining was considered indeterminate unless it was diffusely present. Macrophages, histiocytes, and inflammatory cells served as internal positive controls.

Statistical analysis was performed using SPSS software (version 28; IBM Corp., Armonk, NY, US), with Fisher’s exact test applied to determine the association between galectin-3 expression and the nature of the lesion. A p-value of less than 0.05 was considered statistically significant.

## Results

The data obtained from the 56 thyroid specimens were systematically categorized into the malignant and non-malignant groups based on histopathological evaluation. Galectin-3 expression was assessed through immunohistochemical analysis, and its association with the histological classification was evaluated. The results were presented in terms of frequencies and percentages for categorical variables, allowing a clear comparison between galectin-3 positivity across the two diagnostic groups.

Pre-operative diagnoses varied across the patients. The most frequent pre-operative diagnosis was thyroid malignancy, accounting for 41.07% (n=23) of cases, followed by benign thyroid tumor at 26.79% (n=15). Papillary thyroid malignancy was the pre-operative diagnosis in 21.43% (n=12) of cases, while multinodular goitre was the diagnosis in 8.93% (n=5). Only a small proportion (1.79%; n=1) was sent as solitary thyroid nodules (Table 1).

**Table 1:** Pre-operative diagnosis among the study population

Pre-operative Diagnosis	Number of Cases (n= 56)	Percentage
Benign Thyroid Tumor	15	26.79%
Multinodular Goitre	5	8.93%
Papillary Thyroid Malignancy	12	21.43%
Solitary Thyroid Nodule	1	1.79%
Thyroid Malignancy	23	41.07%

The gross appearance of the thyroid lesions at the time of examination also showed varied distribution. The majority of lesions were described as solitary regular nodules, comprising 48.21% (n=27) of cases. A solitary irregular nodule was observed in 37.50% (n=21). Multiple irregular nodules and multiple regular nodules accounted for 10.71% (n=6) and 3.57% (n=2), respectively.

The histopathological diagnosis revealed an equal proportion of benign and malignant lesions, both accounting for 44.64% (n=25 each) of the cohort. Borderline malignant lesions constituted the remaining 10.71% (n=6).

Classification of thyroid lesions by histopathological diagnosis

The definitive histopathological examination (HPE) diagnosis provided a detailed classification of the thyroid lesions. The most common diagnosis was papillary thyroid carcinoma - classic variant, accounting for 30.36% (n=17) of cases. Follicular adenoma was diagnosed in 25.00% (n=14), and hyperplastic nodule of the thyroid in 19.64% (n=11). Other diagnoses included non-invasive follicular thyroid neoplasm with papillary-like nuclear features (10.71%, n=6), papillary thyroid carcinoma - follicular variant (8.92%, n=5), and encapsulated follicular variant of papillary thyroid carcinoma (3.57%, n=2). A single case of papillary thyroid carcinoma - Warthin-like variant (1.79%, n=1) was also identified.

Association of galectin-3 expression with HPE diagnosis

The study's primary objective of association of galectin-3 expression with the morphological categorization of thyroid lesions yielded a highly statistically significant association (Fisher’s exact test, p < 0.001) (Tables 2-3).

**Table 2:** Galectin-3 expression

Galectin-3 expression	Number of cases (n= 56)	%
Positive	17	30.36%
Negative	39	69.64%

**Table 3:** Association of galectin-3 expression with HPE diagnosis HPE: histopathological examination

HPE diagnosis	Galectin-3 expression				p-value
	Positive		Negative		
	n	%	n	%	
Benign	0	0	25	100	<0.001
Borderline malignant	0	0	6	100	
Malignant	17	68	8	32	
Fisher’s exact test was applied					

A crucial finding was that among the 25 benign lesions identified by HPE, none (0%) exhibited positive galectin-3 expression; all 25 (100%) were negative. Similarly, all 6 borderline malignant lesions (100%) were negative for galectin-3 expression. In stark contrast, among the 25 malignant lesions, 68% (n=17) demonstrated positive galectin-3 expression, while 32% (n=8) were negative.

**Figure 1:**
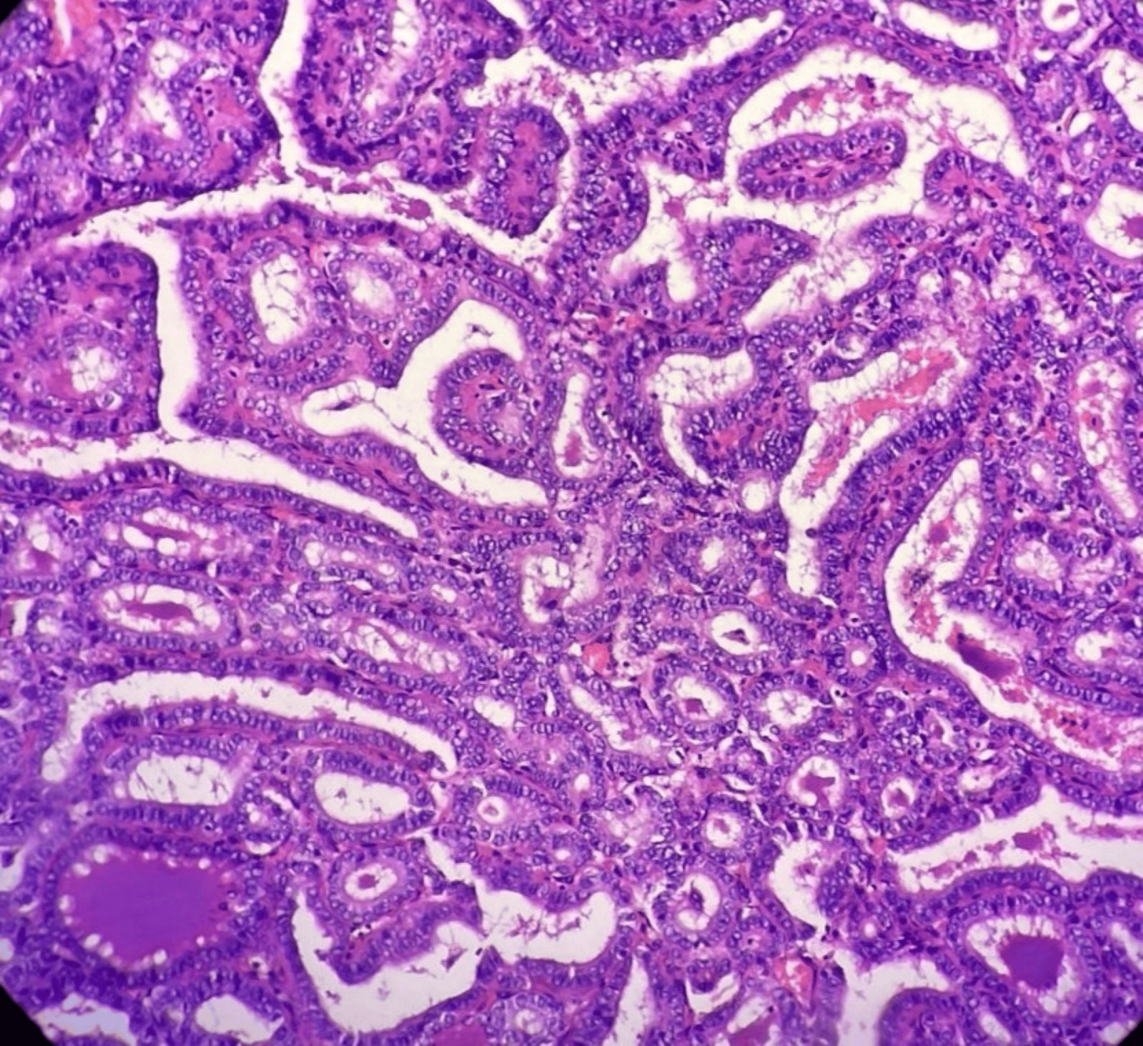
Microphotograph of papillary thyroid carcinoma – classic variant (H&E, 100X)

**Figure 2:**
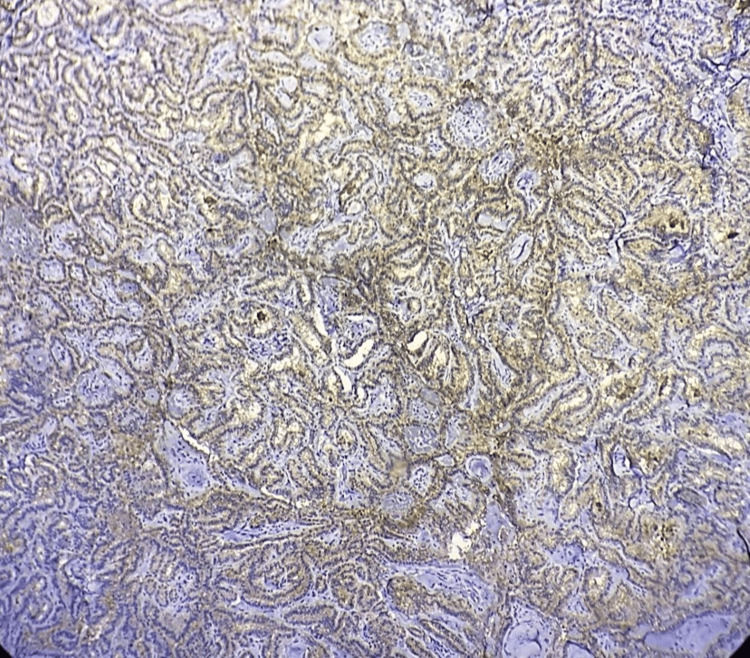
Microphotograph of IHC of galectin-3 showing positive expression in the classic variant of PTC (100X) IHC: immunohistochemistry; PTC: papillary thyroid carcinoma

**Figure 3:**
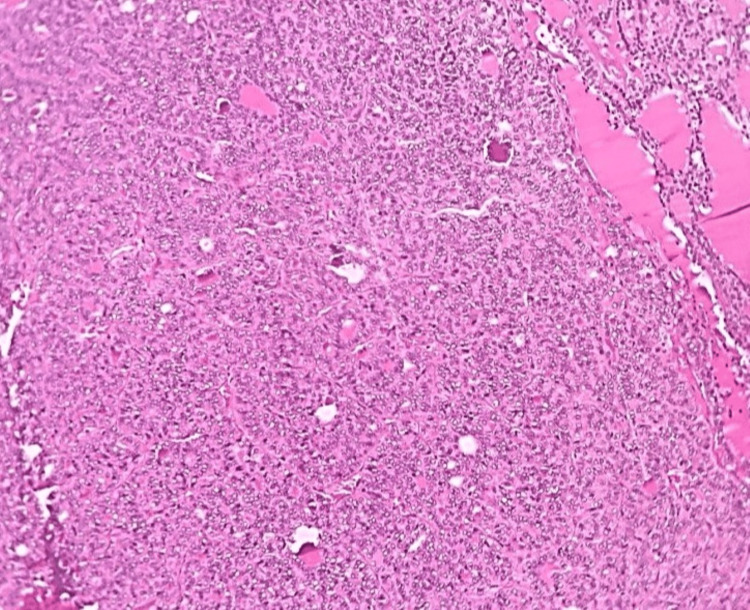
Microphotograph of follicular variant of PTC (H&E,100X) PTC: papillary thyroid carcinoma

**Figure 4:**
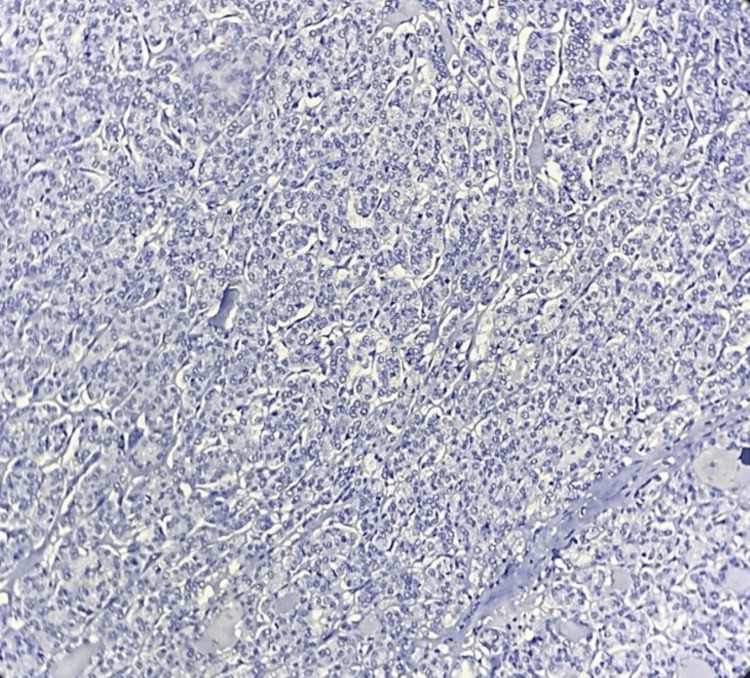
Microphotograph of IHC of galectin-3 showing negative expression in the follicular variant of PTC 100X IHC: immunohistochemistry; PTC: papillary thyroid carcinoma

**Figure 5:**
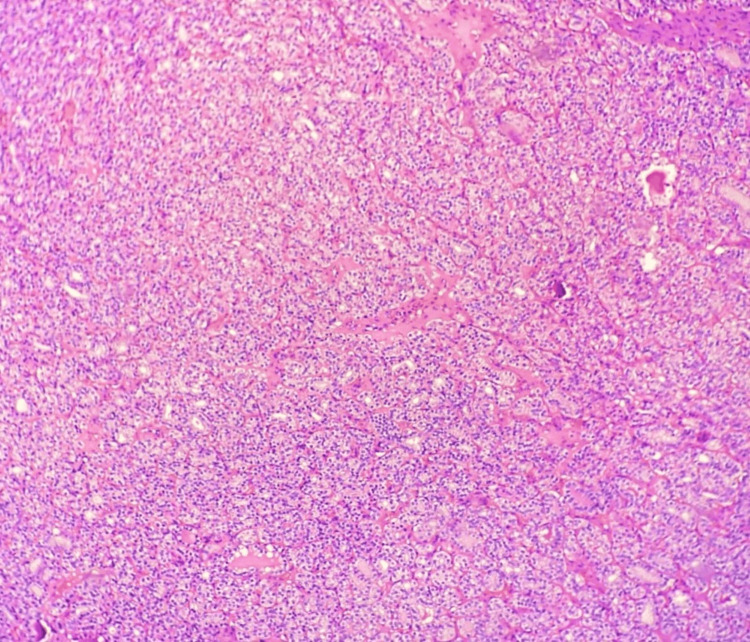
Microphotograph of follicular adenoma of the thyroid (H&E,100X)

**Figure 6:**
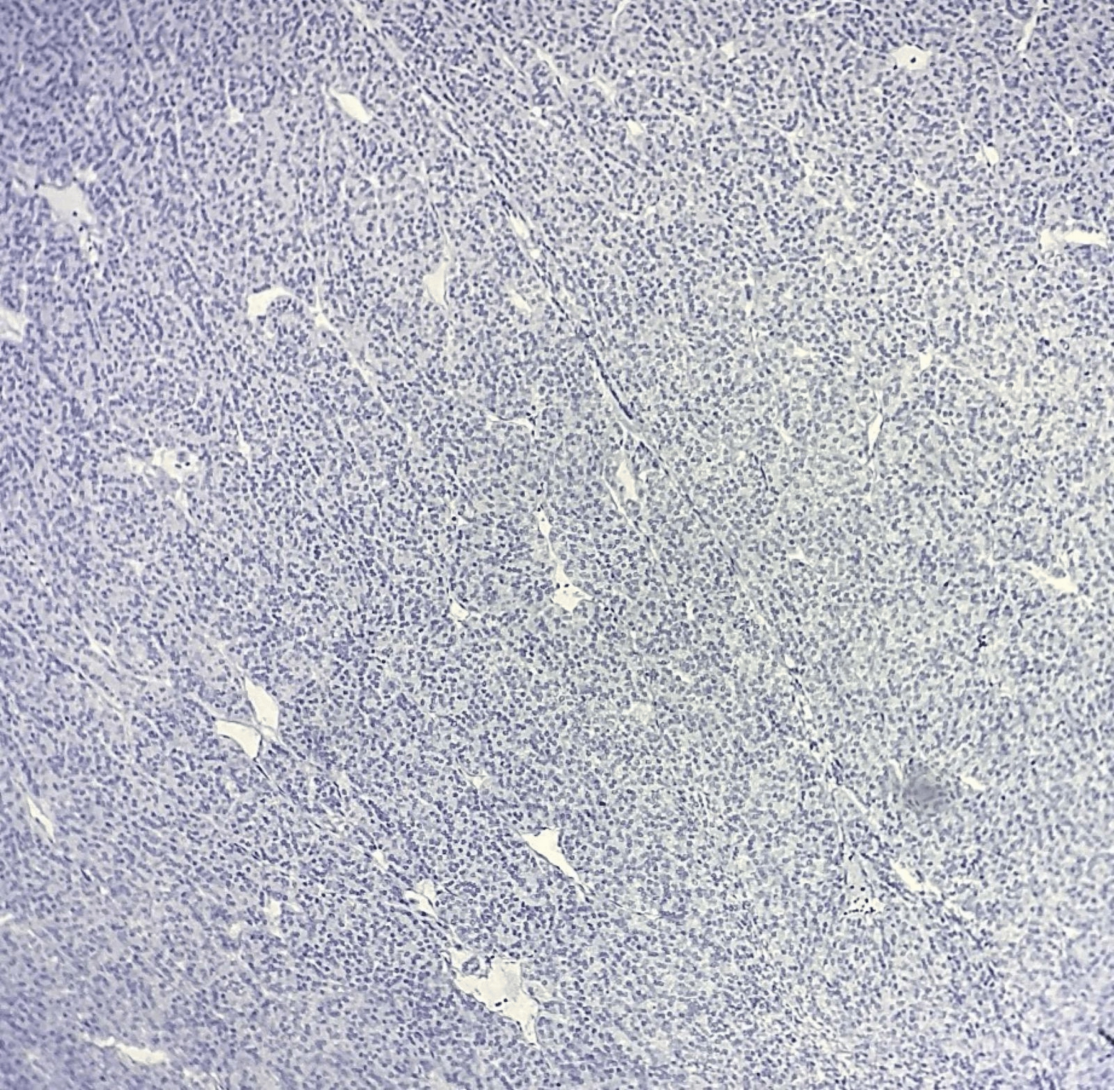
Microphotograph of IHC of galectin-3 showing negative expression in follicular adenoma of the thyroid 100X IHC: immunohistochemistry

Even among the malignancies, all the galectin-3-positive cases belonged to the classic variant of papillary carcinoma. Other malignant lesions, such as follicular variant, encapsulated follicular variant, and Warthin-like variant of papillary carcinoma, were negative for galectin-3 (Table 3, Figures 1-6).

## Discussion

In our study, a total of 56 thyroid specimens with nodular lesions were included, comprising 25 benign, 6 borderline, and 25 malignant lesions diagnosed by histopathology. A statistically significant correlation was observed between galectin-3 expression and malignancy (p < 0.001). Positive galectin-3 staining was noted in 68% (17/25) of malignant lesions, primarily in the classic variant of PTC. None of the benign or borderline lesions, including colloid goitre, follicular adenoma, and non-invasive follicular thyroid neoplasm with papillary-like nuclear features (NIFTP), showed galectin-3 positivity, underscoring its specificity in detecting malignancy.

The method used--immunohistochemistry (IHC)--is consistent with standard diagnostic practices. In our study, galectin-3 expression was limited to the classic variant of PTC; other PTC subtypes did not show positivity. This suggests that galectin-3 should not be used as a standalone marker. Rather, it is recommended to use it in combination with other immunomarkers, such as CD56, CK19, HBME-1, and TPO, especially in indeterminate or follicular-patterned lesions to enhance diagnostic accuracy [15].

Previous studies have reported galectin-3 sensitivity ranging from 86% to 95% and specificity from 85% to 94% in thyroid malignancies. Particularly in cases showing >50% staining positivity, galectin-3 achieves high specificity (up to 100%), reducing the risk of false positives in benign conditions [16]. In indeterminate cytology (Bethesda categories III/IV), galectin-3 has demonstrated a sensitivity of 75% and specificity of 90%, with positive and negative predictive values of 82% and 87%, respectively [10,11].

Galectin-3 exhibits high positivity in PTC (75-95.8%), variable expression in follicular thyroid carcinoma (FTC) (33-75%), and consistent presence in aggressive malignancies like anaplastic and medullary thyroid carcinomas [12]. However, since only classic PTC cases were included in our study, subtype-based comparison was not feasible.

Among other IHC markers, galectin-3 stands out for its diagnostic performance. While HBME-1 and CK19 are also helpful, a panel combining galectin-3 with HBME-1 has been shown to offer optimal diagnostic accuracy, achieving sensitivity and specificity values of 97.3% and 91.2%, respectively, in indeterminate nodules [13,14].

Clinically, galectin-3 integration can reduce unnecessary surgeries. In one study using galectin-3-guided large-needle aspiration biopsy (LNAB), a diagnostic accuracy of 95.3% was achieved, with 70 out of 71 benign cases avoiding surgery [17].

Galectins are also being explored as therapeutic targets in cancer [12]. In thyroid cancer, Ma et al. developed galectin-3-targeted, triple-stimuli-responsive nanoparticles for chemo-photothermal therapy. This enhanced intracellular doxorubicin delivery, reduced cardiac toxicity, and achieved complete tumor eradication in vivo [18]. Menachem et al. combined a Ras inhibitor (FTS) with modified citrus pectin (a Gal-3 inhibitor) to suppress anaplastic thyroid carcinoma, showing effectiveness both in vitro and in vivo by reducing oncogenic signaling and promoting apoptosis [19]. Gheysen et al. used a galectin-1-targeting calixarene derivative, which significantly inhibited proliferation, migration, and metastasis in Gal-1-expressing thyroid cancer cell lines, indicating strong therapeutic potential [20].

For clinical application, a proposed diagnostic algorithm involves initial galectin-3 staining on fine-needle aspiration cytology (FNAC) smears. A staining threshold of ≥50% suggests malignancy and may warrant surgical intervention, while negative staining may support conservative management or the use of additional biomarkers like BRAF or CD56 [9,10].

Several recent studies reinforce galectin-3's diagnostic utility. Soremekun et al. found that 87.5% of malignant tumors were Gal-3 positive, with 82.5% showing strong staining, resulting in a sensitivity of 88% and specificity of 75% [21]. Tang et al.’s meta-analysis confirmed significantly higher Gal-3 expression in PTC, especially in cases with lymph node metastasis [13]. Feilchenfeldt et al. reported elevated Gal-3 mRNA and protein in PTC, though the overlap with follicular adenomas limited the specificity [22]. Sarin et al. observed 100% sensitivity in PTC with strong cytoplasmic staining, while other malignancies showed variable expression [17]. Fu et al. found higher Gal-3 expression in invasive EFVPTC compared to NIFTP, supporting its role in differentiating follicular-patterned lesions [23].

Despite its advantages, the application of galectin-3 is not without limitations. Methodological standardization is essential, as staining protocol variations, antibody clones, and positivity thresholds can affect reproducibility [24]. About 33% of FTCs may not express Gal-3, necessitating the use of adjunct markers. Additionally, rare false positives have been reported in conditions such as Hashimoto’s thyroiditis, where focal staining may occur [25].

Overall, our findings and literature review highlight Galectin-3 as a valuable diagnostic and potentially prognostic marker, particularly in papillary thyroid carcinoma, with promising future applications in targeted therapy.

Strengths and limitations

This study's strengths include its focused evaluation of galectin-3 expression in clearly classified thyroid lesions and the use of a biologically relevant biomarker with a significant correlation to malignancy (p < 0.001), reinforcing its diagnostic utility. However, limitations include the small sample size (n = 56) and the exclusive inclusion of female patients, which may affect generalizability. The absence of interobserver variability analysis may impact the consistency of immunohistochemical interpretation. Additionally, relying solely on galectin-3 without incorporating other complementary markers may limit diagnostic sensitivity in borderline cases.

## Conclusions

In conclusion, this study underscores the utility of galectin-3 as a reliable and specific immunohistochemical marker for the differentiation of malignant from benign thyroid lesions. The absence of galectin-3 positivity in benign and borderline lesions, alongside its high expression in confirmed malignancies, supports its application as a routine component in immunohistochemical panels to enhance diagnostic confidence, particularly in histologically ambiguous or follicular-patterned nodules.

Future studies with larger and more diverse populations, as well as multi-marker approaches and longitudinal follow-up, are warranted to expand the scope of galectin-3 from a diagnostic tool to a potential prognostic and therapeutic biomarker in thyroid oncology.
